# Evaluation of the yeast phase-specific monoclonal antibody 4D1 and *Galanthus nivalis* agglutinin sandwich ELISA to detect *Talaromyces marneffei* antigen in human urine

**DOI:** 10.3389/fcimb.2023.1163868

**Published:** 2023-08-29

**Authors:** Fangyi Shu, Kritsada Pruksaphon, Joshua D. Nosanchuk, Patcharin Thammasit, Sirida Youngchim

**Affiliations:** ^1^ Department of Microbiology, Faculty of Medicine, Chiang Mai University, Chiang Mai, Thailand; ^2^ Department of Anatomy, Youjiang Medical University for Nationalities, Baise, China; ^3^ Department of Medicine (Infectious Diseases), Albert Einstein College of Medicine, Bronx, NY, United States

**Keywords:** sandwich ELISA, *Talaromyces marneffei*, urine, monoclonal antibody (MAb) 4D1, invasive fungal infection, lectin

## Abstract

*Talaromyces* (*Penicillium*) *marneffei* (TM) is an important, but neglected, thermally dimorphic fungus. It is the pathogenic cause of talaromycosis, which is strongly associated with the immunodeficiency state present in individuals with advanced HIV disease. The purpose of this study was to develop a sandwich enzyme-linked immunosorbent assay (sandwich ELISA) for the detection of *T*. *marneffei* cytoplasmic yeast antigen (TM CYA) in human urine. Monoclonal antibody (MAb) 4D1 specifically binds to TM CYA. *Galanthus nivalis* agglutinin (GNA), a mannose -binding lectin, recognizes and binds to mannose residues of TM CYA. For the sandwich ELISA, the microplate was coated with GNA as the capturing molecule for absorbing immune complexes of MAb 4D1-TM CYA. The MAb 4D1-GNA sandwich ELISA did not detect a cross-reaction with other antigens from other fungi or bacteria. Seventy-four urine samples from patients with blood culture -confirmed talaromycosis and 229 urine samples from people without talaromycosis residing in the endemic area were subjected to the MAb 4D1-GNA sandwich ELISA. At an optical density (OD) cutoff value of 0.356, the sensitivity was 89.19% [95% confidence interval (CI): 79.80% –95.22%]; the specificity was 98.69% (95% CI: 96.22% –99.73%). The diagnostic performance of the MAb 4D1-GNA sandwich ELISA was highly consistent with those of blood culture and the Platelia *Aspergillus* galactomannan (GM) ELISA kit. Collectively, the MAb 4D1-GNA sandwich ELISA is a promising technique for the rapid diagnosis of *T*. *marneffei* infection, which would facilitate the early treatment of patients with talaromycosis and it may be used to monitor treatment responses.

## 1 Introduction

The World Health Organization (WHO) has prioritized the issue of invasive fungal infections by releasing the first Fungal Priority Pathogen List (WHO FPPL) in 2022. This list is aimed at guiding research and resource allocation toward the development of new and improved diagnostic tools, treatments, and preventive measures for the most significant fungal pathogens. The positioning of *Talaromyces marneffei* (previously named *Penicillium marneffei*) in the “medium priority group” of WHO FPPL indicates a significant level of public health impact and that there is a need for further investigation into various aspects of talaromycosis, including improving diagnostic tools (Medina et al., 2022; WHO FPPL, 2022). *T. marneffei* is a thermally dimorphic fungus that grows as a mycelial form at 25°C and a binary fission yeast form at 37°C. *T*. *marneffei* is an opportunistic pathogen primarily identified in patients with AIDS, particularly in Southeast Asia and southern China (Wang et al., 2023). *T*. *marneffei* infection is increasingly identified in patients with non-HIV -associated immunodeficiencies, such as systemic lupus erythematosus (SLE), cancer, organ transplantation, and adult-onset immunodeficiency syndromes (Pruksaphon et al., 2020a). The definitive diagnosis of *T*. *marneffei* infection is dependent on the isolation and identification of the fungus from clinical samples by fungal morphology, biochemical testing, and thermal dimorphism (Wong et al., 2001). However, some patients may have a negative blood culture, and the time to positive culture can take up to 14 days (Cao et al., 2019), which is not conducive to early diagnosis and treatment. The use of matrix-assisted laser desorption/ionization time-of-flight mass spectrometry (MALDI-TOF-MS) is promising for reducing the time to identification of *T. marneffei*, but this methodology is not widely available in many regions where talaromycosis occurs (Borman et al., 2019). Numerous studies have been conducted to develop immunodiagnostic assays for *T*. *marneffei* infection. These studies are divided into two types, one for *T*. *marneffei* antigen detection and the other for *T. marneffei* -specific antibody detection in various types of clinical specimens. Specific antibody detection is limited by the impaired immunity of patients with AIDS. Therefore, most investigations have focused on the development of antigen detection assays (Pruksaphon et al., 2020a). In the case of antigen detection, blood (plasma or serum) and urine are suitable samples for clinical diagnosis. The mannoprotein Mp1p is a unique and important component of the *T. marneffei* yeast cell wall and is secreted in large amounts (Cao et al., 1998; Wang et al., 2011; Woo et al., 2016). A novel Mp1p antigen-detecting enzyme immunoassay (EIA) was developed using a mouse monoclonal antibody (MAb) and rabbit polyclonal antibody (Thu et al., 2021), and the Mp1p EIA can be used to detect both urine and plasma to diagnose talaromycosis. Moreover, the Mp1p EIA offered a significantly increased diagnostic sensitivity when the result was paired between plasma and urine testing, increasing sensitivity from 82.9% to 88.8% (Thu et al., 2021). Moreover, the detection of Mp1p antigen testing is superior to that of the galactomannan (GM) assay, especially for patients with low CD4 counts (< 50 cells/mL) (Ly et al., 2020; Chen et al., 2022). This suggests that Mp1p antigen testing based on EIA is more effective for immunocompromised patients and in cases of latent infection.

To develop more sensitive and specific diagnostic assays, a highly specific MAb 4D1 against *T. marneffei* cytoplasmic yeast antigen (TM CYA) was developed in both enzyme-linked immunosorbent assay (ELISA) and lateral flow immunochromatographic formats (LFA) (Prakit et al., 2016; Pruksaphon et al., 2018; Pruksaphon et al., 2020a). The ELISA and LFA are both commonly used for diagnostic formats in the field of clinical immunology, but they have distinct advantages and disadvantages. First, diagnostic sensitivity and lower limit of detection: ELISA generally offers higher sensitivity than LFA. ELISA can detect even low levels of target antigens in a sample due to the signal amplification provided by enzyme-labeled conjugate antibodies. Thus, an ELISA is suitable for detecting small amounts of antigens, which is particularly important when a patient has received an antifungal drug or in the absence of fungemia, as relatively low antigenic levels occur in such patients (Wong et al., 2001; Huang et al., 2007; Pruksaphon et al., 2020a). Second, quantitative analysis: ELISA provides quantitative or semiquantitative results by measuring the antigen concentration (via a standard curve), optical density (OD) index, or cutoff point OD, which is particularly valuable when assessing the progression or severity of disease, patient immune status (CD4 level), or the effectiveness of a therapeutic (Prakit et al., 2016; Chen et al., 2022). 

In the current work, we used MAb 4D1 and *Galanthus nivalis* agglutinin (GNA) to develop a MAb 4D1-GNA sandwich ELISA to enhance the accuracy of quantifying the concentration of TM CYA in urine samples. Microplate wells were coated with GNA, which effectively bound mannose residues of TM CYA in the MAb 4D1-TM CYA immune complex, and the test results demonstrated that the sandwich ELISA was a sensitive and highly specific assay for assessing urine antigen levels. Moreover, we measured *T. marneffei* cell wall GM in the urine samples with the Platelia *Aspergillus* GM commercial kit, and the diagnostic performance of the MAb 4D1-GNA sandwich ELISA was similar to the GM assay (Huang et al., 2007; Zheng et al., 2015).

## 2 Materials and methods

### 2.1 Urine samples

**Figure 1:**
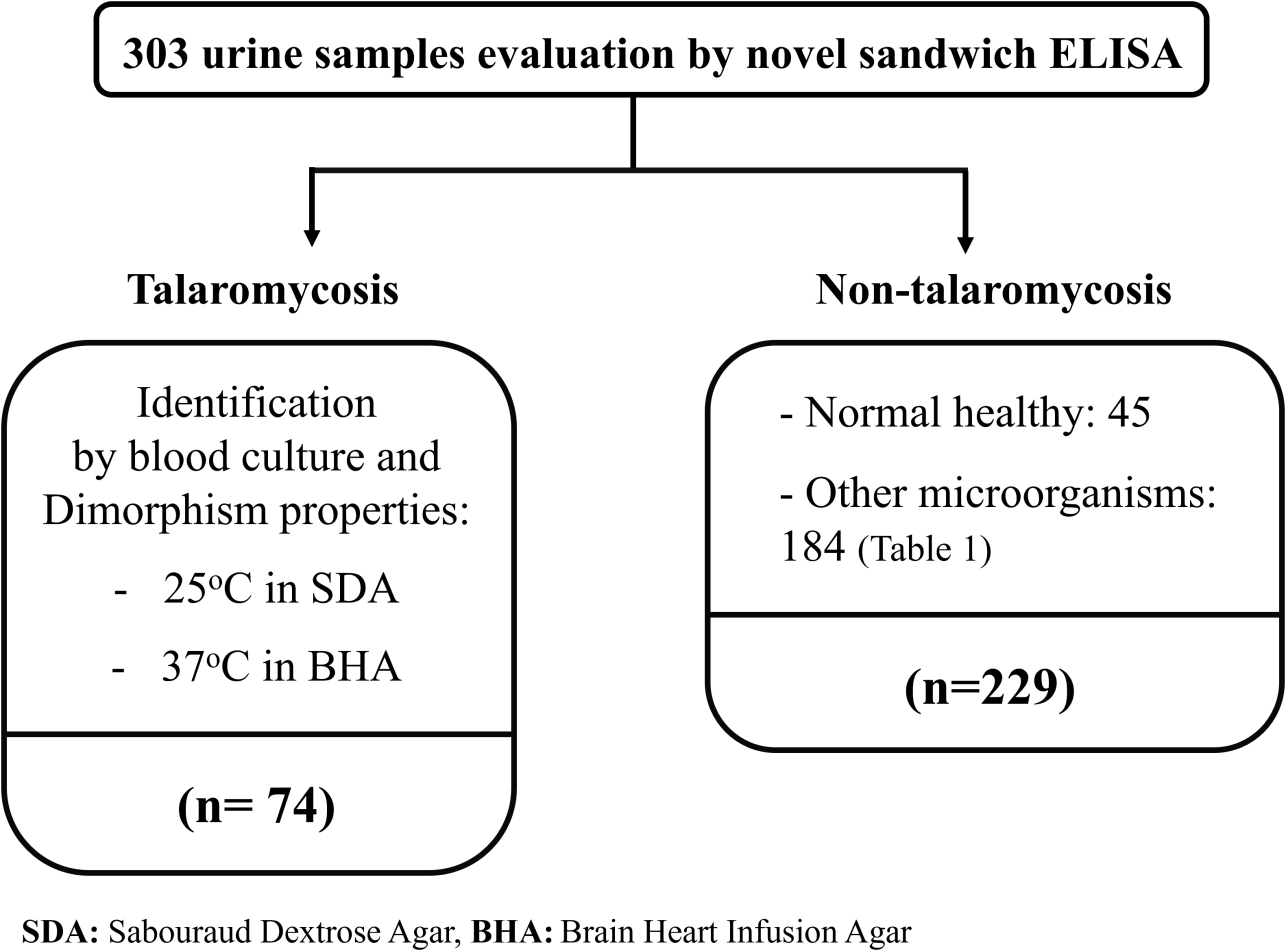
The diagram describes the selection of 303 clinical samples for these studies. The blood culture-proven talaromycosis included 74 cases. Non-talaromycosis controls included 229 participants including 45 normal healthy and 184 of another microorganism infection (classified in **Table 1**). All infected patients were hospitalized at the tertiary hospital of Faculty of Medicine, Chiang Mai University, Chiang Mai, Thailand.

The diagnostic performance of the MAb 4D1-GNA sandwich ELISA was evaluated using 303 clinical urine samples. Seventy-four urine samples were from patients with blood culture-proven talaromycosis. The positive blood cultures were confirmed by thermal dimorphic transition between 25°C and 37°C and microscopic analysis for phenotypic characterization. One hundred eighty-four urine samples were from patients infected with other pathogenic microorganisms (e.g., fungus, virus, parasite, or bacterium). Forty-five urine samples were from healthy individuals living in our endemic area. The samples from patients infected with other pathogenic microorganisms and healthy individuals acted as the control group. All urine samples were collected from September 2004 to September 2019 at the clinical pathology unit, tertiary hospital of Chiang Mai University (Maharaj Nakorn Chiang Mai Hospital), Chiang Mai, Thailand (**Figure 1**). The samples were stored at -80°C until use. The Research Ethics Committee of the Faculty of Medicine, Chiang Mai University, approved this study (Study code: MIC-2565-09250). The sample sizes were estimated based on the anticipated sensitivity and specificity (Buderer, 1996). The parameters were established as follows: the error rate α = 0.05, e = 0.1 (10%), confidence (1-α) = 95%, expected sensitivity = 0.9, expected specificity = 0.9 (Hajian-Tilaki, 2014). The minimum acceptable sampling size was 70 for both talaromycosis (case) and non- talaromycosis (control) when calculated without the dropout rate. Additionally, urine samples from patients infected with other pathogenic microorganisms are detailed in **Table 1**.

**Table 1:** Urine samples from patients infected with other pathogenic microorganisms (non-talaromycosis control).

Urine samples	No.	Urine samples	No.	Urine samples	No.
*Cryptococcus neoformans*	14	*Histoplasma capsulatum*	2	*Candida albicans*	12
*Candida tropicalis*	6	*Candida glabrata*	7	*Candida krusei*	5
*Candida guilliermondii*	1	*Candida parapsilosis*	1	Diabetes mellitus with candidiasis	3
*Geotrichum spp*.	3	*Trichosporon spp.*	3	*Fusarium keratoplasticum*	1
*Pneumocystis jirovecii pneumonia*	1	Unidentified budding yeast cell with pseudohyphae	13	Serum galactomannan positive patients	5
Tuberculosis	5	*Staphylococcus aureus*	2	Coagulase-negative staphylococci	4
*Streptococcus pyogenes*	1	*Streptococcus suis*	1	*Streptococcus pneumoniae*	2
*Enterococcus faecium* with unidentified budding yeast cell	3	*Enterococcus faecalis*	7	*Escherichia coli* (ESBL producing)	10
*Klebsiella pneumonia* (ESBL producing)	8	*Citrobacter freundii*	2	*Morganella morganii*	4
*Proteus vulgaris*	4	*Proteus mirabilis*	3	*Serratia marcescens*	4
*Salmonella* sp.	1	*Acinetobacter baumannii*	13	*Pseudomonas aeruginosa*	11
*Stenotrophomonas maltophilia*	3	Herpes simplex virus	3	Hepatitis B virus	7
Hepatitis C virus	6	Toxoplasmosis	3	**Total**	**184**

### 2.2 *T. marneffei* cytoplasmic yeast antigen preparation

The TM CYA preparation was performed as described (Prakit et al., 2016). Briefly, *T*. *marneffei* conidia were inoculated into brain heart infusion (BHI) broth **(**Becton Dickinson, Sparks, MD, USA) and cultured for 6 days at 37**°**C, 150 rpm. After treatment with 0.02% (w/v) Merthiolate (Sigma, Poole, UK) at room temperature for 12 h, the yeasts were centrifuged for 10 min at 2,300 g and the cell walls were mechanically broken with 0.5-mm glass Ballotini beads (BioSpec, Bartlesville, OK, USA) in a bead beater homogenizer (BioSpec, Bartlesville, OK, USA). A cocktail of protease inhibitors including iodoacetic acid (IAA; 10 μM**;** Sigma), phenylmethanesulfonyl fluoride (PMSF; 0.1 mM**;** Sigma), and 1 mM ethylene diamine tetraacetic acid (EDTA), was added to prevent fungal protein degradation. The mixture was centrifuged for 30 min at 4**°**C at 10,000 g, and the total antigen was collected. The antigens of other fungi were prepared following the same standardized procedure. The fungal strains used in the current study are listed in **Table 2**.

**Table 2:** Fungal isolates.

Fungal species	Isolate number
*Talaromyces marneffei*	ATCC 200051^#^
*Cryptococcus neoformans*	H99^#^
*Candida albicans*	ATCC 900028^#^
*Sporothrix schenckii*	52-S1*
*Histoplasma capsulatum*	53-H1*
*Aspergillus fumigatus*	55-A1*
*Penicillium citrinum*	MMC59P12-1^$^
*Pseudallescheria boydii*	MMC60S21-1^$^
*Geotrichum* spp.	CI
*Candida krusei*	CI
*Trichosporon* spp.	CI

^#^Isolate from the American Type Culture Collection, Rockville, MD, USA.

*Isolates from the Institute of Dermatology, Department of Medical Services, Ministry of Public Health, Bangkok, Thailand.

^$^Isolate from the culture collection in the Mycology Unit, Department of Microbiology, Faculty of Medicine, Chiang Mai University, Chiang Mai, Thailand.

CI, clinical isolates from blood samples of infected patients.

### 2.3 Purification of MAb 4D1

The murine-derived hybridoma cell line 4D1 was cultured in hybridoma serum -free medium (Gibco). The culture supernatant of MAb 4D1 was concentrated with a Vivaspin 20 ultrafiltration device (MWCO: 30 kDa). The supernatant was filtered through a 45 -µm membrane filter. Subsequently, the concentrated 4D1 supernatants were purified by HiTrap protein G affinity chromatographic column (GE Healthcare). The protein G was covalently linked to Sepharose bead and captured MAb 4D1, which belongs to the mouse IgG1 subclass. The MAb 4D1 was eluted by 0.1 M glycine-HCl pH 2.7 and was immediately neutralized by adding 1 M Tris-HCl pH 9.0. Subsequently, the eluted fractions, not containing MAb 4D1, were screened out by indirect ELISA. The eluted fractions containing MAb 4D1 were pooled and diafiltrated in PBS pH 7.4 and then diafiltrated again with 20 mM sodium phosphate buffer pH 7.2. The MAb 4D1 concentrations were determined by spectrophotometry at 280 nm (NanoDrop, Thermo Scientific) and were calculated by the molar extinction coefficient at 1 absorbance unit of purified murine IgG (1.36 for a solution of 1 mg/mL) (Pruksaphon et al., 2021). The purity of MAb 4D1 was validated using 10% SDS-PAGE. To confirm the mouse IgG subclass of MAb 4D1 by immunoblotting, the SDS-PAGE gel containing separated polypeptides was transferred electrophoretically to nitrocellulose membranes (Hybond extra, Amersham). The membranes were washed with PBS and then blocked overnight at 4°C with PBS-0.05% Tween 20 containing 5% skim milk (Sigma). Later, the membranes were washed with PBS-0.05% Tween 20 and incubated for 60 min with HRP -conjugated goat anti-mouse IgG (Jackson, West Grove, PA, USA) diluted 1:1,000 in PBS-0.05% Tween 20 containing 1% skim milk. The membranes were washed, and the bound conjugate was developed and visualized by incubation with TMB/H_2_O_2_ chromogenic substrate (BioFX Laboratories SurModics). The reactions were stopped by submersion of the membrane in distilled water (Prakit et al., 2016).

### 2.4 Detection of the specificity, construction of the standard curve, and sample detection

The purified GNA lyophilized powder from snowdrop (*G. nivalis*) bulbs was solubilized according to the manufacturer**’**s instructions (Sigma). To detect the specificity and construct the standard curve of the MAb 4D1-GNA sandwich ELISA, 100 μL of TM CYA or other fungal antigens (0.1953 –100 µg/mL) was spiked into the urine of a healthy individual and mixed with 100 μL of MAb 4D1 (1 µg/mL). For sample detection, 100 μL of urine was mixed with an equal volume of MAb 4D1 (1 µg/mL). The mixtures were incubated for 90 min at 25**°**C, 200 rpm, and then at 4**°**C overnight for immune complex formation. Fifty microliters of 12.5 µg/mL purified GNA in 0.06 M carbonate coating buffer (pH 9.6) was added to wells of a MaxiSorp 96-well microtiter plate (Nunc A/S). The plate was incubated for 80 min at 37**°**C and then washed five times with phosphate buffer saline (pH 7.2) containing 0.05% Tween 20 (PBST). The wells were blocked by 220 µL of 3% (w/v) bovine serum albumin (BSA; Bio Basic, Konrad Crescent, Markham, ON, Canada) in PBS at 4**°**C overnight. After washing, 50 µL of immune complex was added to the wells in triplicate and incubated for 60 min at 25**°**C, 300 rpm. Then, the plate was washed five times with PBST. Fifty microliters of HRP -conjugated goat anti-mouse IgG (Jackson, West Grove, PA, USA) diluted 1:10,000 in diluting buffer (0.1% w/v BSA in PBST) was added followed by 60 -min incubation at 25**°**C, 300 rpm. After washing, 100 µL of 3,3**’**,5,5**’**-tetramethylbenzidine (TMB; Surmodics IVD, Eden Prairie, MN, USA) was added, and the plate was incubated for 10 min at room temperature in the dark. After stopping the reaction by adding 50 µL of 2** N** H_2_SO_4_, the absorbance was measured on an ELISA reader (Shimadzu model UV-2401PC, Kyoto, Japan) at 450 nm with wavelength correction set at 570 nm.

### 2.5 Detection of galactomannan in urine samples

GM in urine samples was measured with the commercial Platelia *Aspergillus* GM ELISA kit (Bio-Rad Laboratories, Redmond, WA, USA) following the manufacturer**’**s instructions. Briefly, 300 μL of urine (negative, positive, and cutoff) was treated with 100 μL of sample treatment solution. Then, the mixtures were boiled for 3 min at 100**°**C and centrifuged for 10 min at 10,000 g. The supernatants were used for the detection of the GM antigen. Fifty microliters of conjugate and 50 μL of supernatant were successively added to the wells in duplicate. After incubation for 90 min at 37**°**C, the plate was washed five times. Two hundred microliters of TMB was added to each well, and the plate was incubated for 25 min in the dark. After stopping the reaction by the addition of 1 N H_2_SO_4_, the absorbance was measured on an ELISA reader at 450 nm with the wavelength correction set at 620 nm. In the current study, a sample with an index ≥ 0.5 was considered positive for GM antigen.

### 2.6 Statistical analysis

The distribution of OD between cases (talaromycosis) and controls (non- talaromycosis) was compared using Mann–Whitney U test (Wilcoxon rank sum test). The receiver operating characteristic (ROC) curve was constructed, which represented all diagnostic specificity and sensitivity pairs for different OD cutoff points using Epitools (Greiner et al., 2000). The differentiation power between cases and controls was defined by calculating the area under the curve (AUC) with 95% confidence interval (95% CI), and the cutoff point OD was established based on the Youden**’**s index on the ROC curve. The diagnostic performance of the MAb 4D1-GNA sandwich ELISA was compared with that of blood culture and the Platelia *Aspergillus* GM ELISA by McNemar test and Cohen**’**s kappa coefficient (Landis and Koch, 1977). All statistical analyses were carried out using SPSS v. 17.0 and GraphPad Prism 8.4.0 with a significant value of *p* < 0.05.

## 3 Results

### 3.1 Purity of the MAb 4D1

**Figure 2:**
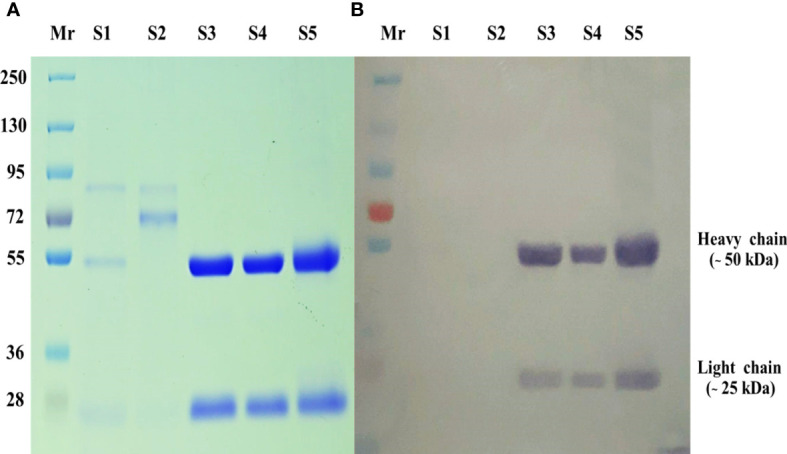
SDS-PAGE of MAb 4D1 purified and concentrated by HiTrap Protein G HP affinity chromatography column. **(A)** SDS-PAGE carried out under reduced conditions (10 µg). **(B)** Determination of mouse IgG isotype of MAb 4D1 by immunoblotting with goat anti-mouse IgG antibody (у chain, κ and λ chain specific). The numbers on the left were relative molecular weights of pre stained markers: Lane Mr, prestained molecular weight markers; Lane S1, hybridoma culture supernatant of MAb 4D1; Lane S2, concentrated hybridoma cultured supernatant of MAb 4D1; Lanes S3 and S4, pooled eluted fractions showing both IgG subunits with molecular weights of 50 kDa (heavy chain) and 25 kDa (light chain); Lane S5, positive control (previous lot of MAb 4D1 described in Pruksaphon et al., 2018).

The purity of the MAb 4D1 fractions was demonstrated by SDS-PAGE (**Figure 2A**). The IgG subclass of MAb 4D1 was studied by immunoblotting with HRP -conjugated goat anti-mouse IgG, which confirmed MAb 4D1 as an IgG1 (**Figure 2B**). The total protein concentration of the purified MAb 4D1 was 0.9 mg/mL.

### 3.2 Specificity and standardization of the MAb 4D1-GNA sandwich ELISA

**Figure 3:**
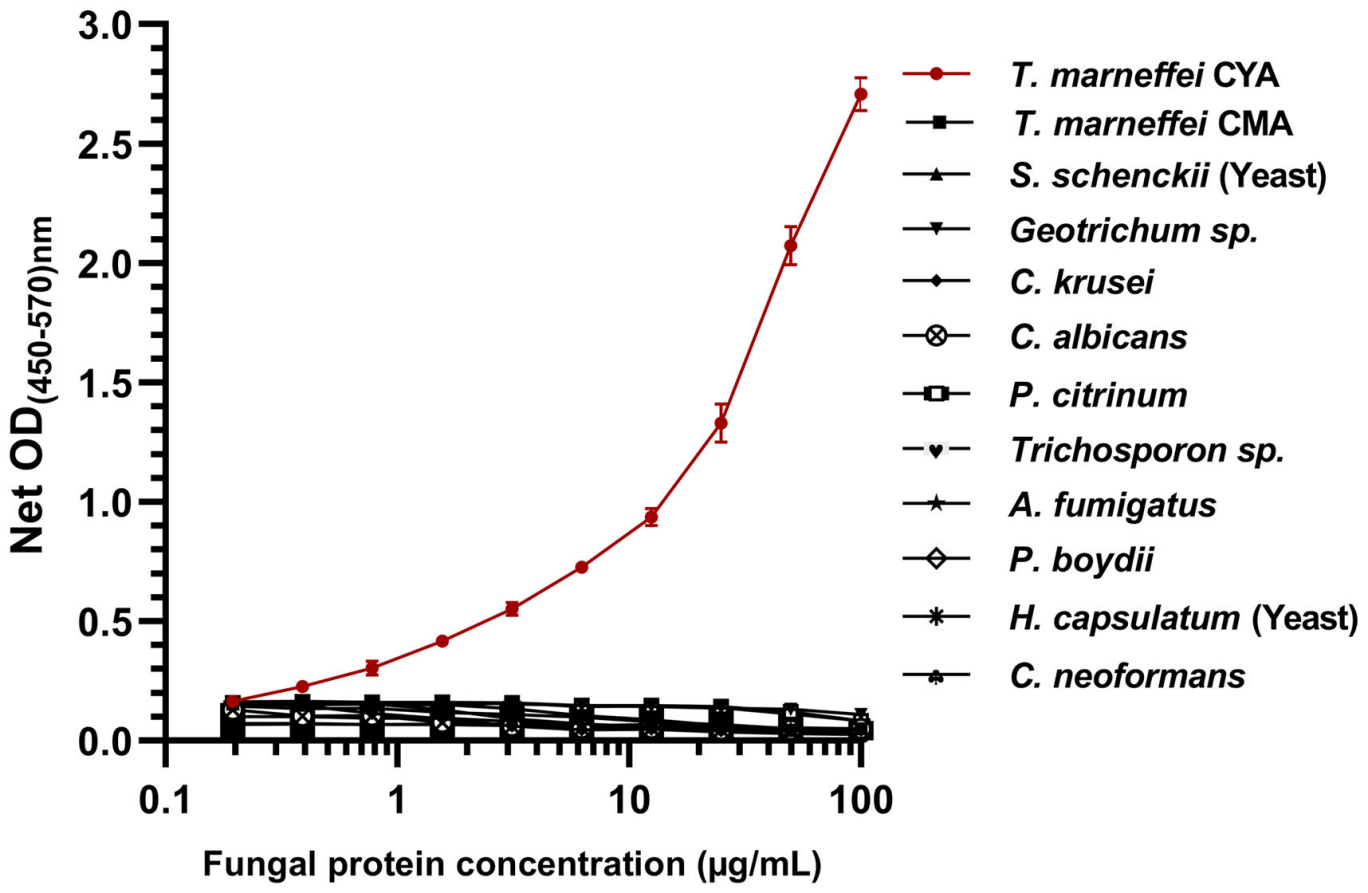
The specificity of the MAb 4D1-GNA sandwich ELISA and the standard curve in the detection of TM CYA. In total, 12 cytoplasmic fungal proteins from 11 clinically important fungi were determined. The concentrations of fungal proteins were log-transformed. The reactivity of TM CYA generated the standard curve. The experiments were carried out in triplicate, and the data were represented as mean ± SEM. CYA, cytoplasmic yeast antigen; CMA, cytoplasmic mycelium antigen.

In this study, we assessed the reactivity of fungal proteins in the MAb 4D1-GNA sandwich ELISA system (**Figure 3**). TM CYA was effectively measured in a concentration -dependent manner using the system, and there was no detectable cross-reactivity to cytoplasmic protein from the control fungi. This result indicated that the MAb 4D1-GNA sandwich ELISA system was highly specific to TM CYA. A standard curve was constructed by the reactions of TM CYA in the system at concentrations ranging from 0.1953 to 100 µg/mL.The standard curve was highly reproducible. At concentrations of 0.1953 –50 µg/mL, the correlation coefficient (*r*) between the OD value and TM CYA concentration was 0.98.

### 3.3 Detection of urine samples by the MAb 4D1-GNA sandwich ELISA and diagnostic performance

**Figure 4:**
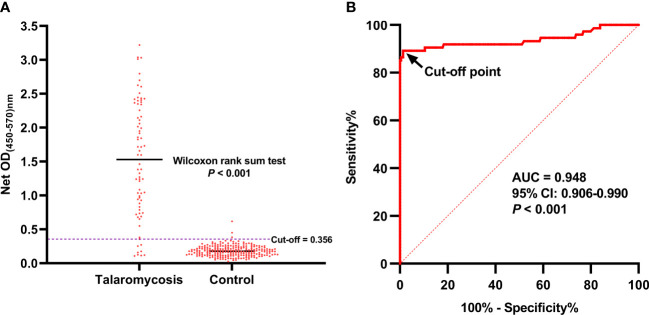
The OD values of the urine samples from patients with talaromycosis and control individuals and ROC curve analysis of the MAb 4D1-GNA sandwich ELISA. The OD values of the two groups were compared using Wilcoxon rank sum test **(A)**. The cutoff was chosen by the Youden index on the ROC curve **(B)**. AUC, area under the ROC curve.

In total, 303 urine samples were subjected to the MAb 4D1-GNA sandwich ELISA, including 74 samples from patients with talaromycosis and 229 samples from the control group without talaromycosis. The OD values of the talaromycosis group and the control group were significantly different (**Figure 4A**). This result implied that MAb 4D1 was specifically reactive to TM CYA in urine, allowing the MAb 4D1-GNA sandwich ELISA to distinguish patients with talaromycosis from controls. The ROC curve was constructed by plotting sensitivity (true positives) versus the corresponding 1-specificity (false positives) at various OD cutoff values (**Figure 4B**). The area under the ROC curve (AUC) is an important index of accuracy of the curve (DeLong et al., 1988; Trifonova et al., 2013).

In this study, the AUC was 0.948 (95% CI: 0.906 –0.990, *p* < 0.001), which indicated that the MAb 4D1-GNA sandwich ELISA offered excellent detectability of talaromycosis. The operating point in the upper left corner of the curve indicated by the arrow was chosen as the cutoff with an OD value of 0.356. The corresponding diagnostic performance was illustrated in **Table 3**. The MAb 4D1-GNA sandwich ELISA correctly diagnosed talaromycosis in 66 of 74 (89.19%) cases (95% CI, 79.80% –95.22%), suggesting no significant difference compared to the blood culture assay (*p* > 0.05). The test correctly excluded talaromycosis in 226 of 229 (98.69%) control individuals (95% CI, 96.22% –99.73%). The accuracy was 96.37% (95% CI, 93.60% –98.17%). The positive likelihood ratio (LR) was 68.08, indicating an association with disease. The negative LR was 0.11, suggesting an association with the absence of disease. Overall, there was a practically perfect agreement between the MAb 4D1-GNA sandwich ELISA and the blood culture assay (kappa = 0.899, 95% CI: 0.841–0.958, *p* < 0.001) (Landis and Koch, 1977).

**Table 3:** Diagnostic performance of the MAb 4D1-GNA sandwich ELISA when testing urine samples from individuals with or without talaromycosis.

Performance parameter	MAb 4D1-GNA sandwich ELISA	
	Value	95% CI*
Sensitivity	89.19%	79.80% - 95.22%
Specificity	98.69%	96.22% - 99.73%
Accuracy	96.37%	93.60% - 98.17%
Positive likelihood ratio	68.08	22.06 - 210.12
Negative likelihood ratio	0.11	0.06 - 0.21
kappa	0.899	0.841 - 0.958

* Confidence interval.

### 3.4 Comparison of the MAb 4D1-GNA sandwich ELISA with Platelia Aspergillus GM ELISA

All urine samples were also tested with the Platelia *Aspergillus* GM ELISA kit. The diagnostic performance of the MAb 4D1-GNA sandwich ELISA was compared with that of the Platelia *Aspergillus* GM ELISA (**Table 4**). There was a virtually perfect agreement between the two assays (kappa = 0.906, 95% CI: 0.849 –0.963, *p* < 0.001). This result was confirmed by the McNemar test (*p* > 0.05).

**Table 4:** Two-by-two table analysis of the diagnostic performance of the MAb 4D1-GNA sandwich ELISA and the Platelia sandwich ELISA.

MAb 4D1-GNA sandwich ELISA	Platelia *Aspergillus* GM ELISA		Total
	Positive	Negative	
Positive	64	5	69
Negative	5	229	234
Total	69	234	303

## 4 Discussion

Talaromycosis is traditionally diagnosed by identification of *T*. *marneffei* from clinical specimens by microscopy or culture. A rapid presumptive diagnosis of talaromycosis can be achieved by microscopic examination of skin or node biopsy specimens. However, for example, in Northern Thailand, only 40.5% of patients presented with skin lesions and 31.9% with lymphadenopathy (Kawila et al., 2013). Mycological culture is the gold standard method for the diagnosis of *T. marneffei* infection, but it is more time-consuming, as it usually takes 3 –14 days for growth to be detected (Cao et al., 2019). Delay in diagnosis negatively affects the initiation of the appropriate treatment. The development of assays for rapid diagnosis of talaromycosis is urgently needed. MAb 4D1 is highly specific against TM CYA (Prakit et al., 2016; Pruksaphon et al., 2020b). Therefore, MAb 4D1 is a promising tool that can be used for developing rapid and specific diagnostic assays for the identification of *T. marneffei* infection. In the current study, we developed a MAb 4D1-GNA sandwich ELISA for the diagnosis of *T. marneffei* infection using urine. We demonstrated that the system is specific, in a concentration -dependent manner, for *T. marneffei*, as there was no cross -reaction of MAb 4D1 with antigens of other fungi (**Figure 3**). At the concentration of 0.1953–50 µg/mL, the correlation coefficient (*r*) between concentration and OD value was 0.980. This result confirmed the high specificity of MAb 4D1 to TM CYA in urine using the MAb 4D1-GNA sandwich ELISA system. In addition, the concentration of TM CYA in the urine can be determined by the MAb 4D1-GNA sandwich ELISA. In future studies, we will attempt to select other species of clinical relevance, including other *Talaromyces* species such as *Talaromyces amestolkiae*, *Talaromyces purpurogenus*, *Talaromyces atroroseus*, and *Talaromyces stipitatus* (Li et al., 2019) to test the specificity of this ELISA format.

We examined 303 urine samples with the MAb 4D1-GNA sandwich ELISA, including 74 urine samples from patients with talaromycosis and 229 from controls. The MAb 4D1-GNA sandwich ELISA presented excellent detectability of *T*. *marneffei* antigen in urine (**Figure 4**), and the results of ELISA were highly consistent with those of blood culture assay (**Table 3**) and the Platelia *Aspergillus* GM ELISA. In terms of diagnostic time, the MAb 4D1-GNA sandwich ELISA was significantly shorter than that required for blood culture, which takes up to 14 days for a definitive diagnosis. In our method, the MAb 4D1-GNA sandwich ELISA takes approximately 17 h to complete, and it can be further optimized to reduce this time.

The Platelia *Aspergillus* GM EIA is the alternative serodiagnosis for talaromycosis in many clinical laboratories in endemic areas. The Platelia *Aspergillus* EIA test can be used to diagnose talaromycosis with acceptable diagnostic performances (Huang et al., 2007; Wang et al., 2015; Zheng et al., 2015). The GM antigens between *T. marneffei* and *Aspergillus* sp. share similar antigenicity (Huang et al., 2007). However, several reports have shown significant cross -reactivity of rat MAb EB-A2 against GM antigen from non-*Aspergillus* species, particularly with *Geotrichum capitatum*, *Histoplasma capsulatum*, *Paracoccidioides brasiliensis*, *Blastomyces dermatitidis*, *Penicillium* sp., and *Cryptococcus neoformans*. Therefore, the GM EIA lacks specificity, as it was unable to distinguish between *Aspergillus* sp. and other pathogenic fungi including *T. marneffei* (Pruksaphon et al., 2020a). Additionally, it is relatively cost-prohibitive to use in resource -poor regions.

The presence of a specific fungal antigen or some metabolites in urine samples from infected patients with histoplasmosis, blastomycosis, paracoccidioidomycosis, and talaromycosis has been used for diagnostic purposes (Thu et al., 2021; Almeida-Paes et al., 2022 **).** Our MAb 4D1-GNA sandwich ELISA is inexpensive and easy to perform, and the urine samples are collected noninvasively. Therefore, we believe that our assay in urine samples has the potential to remarkably improve the management of talaromycosis patients. However, the drawback of the MAb 4D1-GNA sandwich ELISA is that it may occasionally produce false -negative or false -positive results. Therefore, when using the MAb 4D1-GNA sandwich ELISA to diagnose talaromycosis, signs and symptoms as well as a history of exposure to *T. marneffei* should be considered. Overall, the inhibition enzyme-linked immunosorbent assay (inh-ELISA) and lateral flow immunochromatographic test (ICT) were developed incorporating MAb 4D1 for the detection of *T. marneffei* infection. The inh-ELISA was used for the detection of diluted serum, while the ICT was suitable for the detection of urine (Prakit et al., 2016; Pruksaphon et al., 2021). Whether the MAb 4D1-GNA sandwich ELISA described in this study can be used for the detection of serum or plasma remains to be further investigated.

There are limitations to this study. First, false -negative urine samples from patients with talaromycosis were stored for years, which may have resulted in peptide antigen degradation. Degradation of the antigen would decrease the OD value, which may reduce the sensitivity of the assay. One potential issue could be that patients may have been in an early stage of infection **(**non-fungemic talaromycosis**)** with negative blood cultures, resulting in their classification into the control negative, causing a false -positive result by MAb 4D1-GNA sandwich ELISA. Also, only a single -time urine sample was collected for each patient with talaromycosis, and the effect of antifungal treatment on OD values was not tracked. Therefore, further investigation is required to determine whether there are changes in OD values with antifungal therapy. Being able to monitor the effect of antifungal treatment on the OD values would play an important guiding role in clinical therapy, and tracking of antigenuria would be a powerful tool for following the efficacy of therapy in patients infected with *T*. *marneffei*. Moreover, all urine samples were collected from patients with a positive hemoculture of *T. marneffei*; the sensitivity of the MAb 4D1-GNA sandwich ELISA was 89.19%. Indeed, approximately 50%–70% of patients with talaromycosis are hemoculture positive (Kawila et al., 2013; Narayanasamy et al., 2021). Thus, the diagnostic sensitivity of such ELISA format might be lower than that in the case of urine samples from talaromycosis patients with negative hemoculture or non-fungemia cases. Finally, when collecting urine samples, the details of the patients were not completely recorded, such as gender, age, and history of antifungal treatment. Nevertheless, our study is an important next step in the exploration of the MAb 4D1-GNA sandwich ELISA for the rapid diagnosis of talaromycosis, which would contribute to the timely antifungal treatment of patients. In future work, the prospective trialing of the MAb 4D1-GNA sandwich ELISA using urine and blood is necessary to fully describe the utility of the system in real-world clinical studies.

## 5 Conclusions

MAb 4D1 is highly specific against TM CYA. The MAb 4D1-GNA sandwich ELISA presented excellent detectability of *T. marneffei* antigen in urine of patients with talaromycosis, and the results were highly consistent with those of blood culture and the Platelia *Aspergillus* GM ELISA. The MAb 4D1-GNA sandwich ELISA was significantly superior to gold standard culture in diagnostic time, which would facilitate the early diagnosis of talaromycosis. Therefore, the MAb 4D1-GNA sandwich ELISA is a promising assay for the diagnosis of talaromycosis.

## Data availability statement

The original contributions presented in the study are included in the article/supplementary material. Further inquiries can be directed to the corresponding author.

## Ethics statement

The study was approved by the Research Ethics Committee of Faculty of Medicine, Chiang Mai University, Thailand. The studies were conducted in accordance with the local legislation and institutional requirements. The ethics committee/institutional review board waived the requirement of written informed consent for participation from the participants or the participants’ legal guardians/next of kin because All clinical samples used in this study were received from an already-existing sample collection in previous studies with specific permission. All samples were anonymized and the study was carried out in compliance with protocols approved by the Research Ethics Committee of Chiang Mai University, Chiang Mai, Thailand (no. MIC-2565-09250).

## Author contributions

Conceptualization, KP and SY; methodology, FS and KP; validation, FS, KP, and SY; formal analysis, FS and KP; investigation, FS, PT, and KP; resources, SY; data curation, FS, KP, JN, PT, and SY; writing—original draft preparation, FS; writing—review and editing, JN, PT, and SY; visualization, KP, PT, and JN; supervision, JN and SY; project administration, SY; funding acquisition, FS and SY. All authors have read and agreed to the published version of the article.
